# High-serum MMP-8 levels are associated with decreased survival and systemic inflammation in colorectal cancer

**DOI:** 10.1038/s41416-018-0136-4

**Published:** 2018-05-29

**Authors:** Päivi Sirniö, Anne Tuomisto, Taina Tervahartiala, Timo Sorsa, Kai Klintrup, Toni Karhu, Karl-Heinz Herzig, Jyrki Mäkelä, Tuomo J. Karttunen, Tuula Salo, Markus J. Mäkinen, Juha P. Väyrynen

**Affiliations:** 1 Cancer and Translational Medicine Research Unit, University of Oulu, POB 5000, 90014 Oulu, Finland; 2 Oulu University Hospital and Medical Research Center Oulu, POB 21, 90029 Oulu, Finland; 3 Department of Oral and Maxillofacial Diseases, University of Helsinki, Haartmaninkatu 8, POB 63, 00014 Helsinki, Finland; 4 Research Unit of Surgery, Anesthesia and Intensive Care, University of Oulu, POB 5000, 90014 Oulu, Finland; 5 Research Unit of Biomedicine and Biocenter Oulu, Department of Physiology, University of Oulu, POB 5000, 90014 Oulu, Finland; 6 Department of Gastroenterology and Metabolism, Poznan University of Medical Sciences, ul. Szpitalna 27/33, 60-572 Poznan, Poland; 7 Helsinki University Hospital, 00014 Helsinki, Finland

**Keywords:** Prognostic markers, Colorectal cancer, Tumour biomarkers, Tumour immunology

## Abstract

**Background:**

Matrix metalloproteinase-8 (MMP-8) is a protease mainly expressed by neutrophils that cleaves numerous substrates, including collagens and cytokines. We have previously shown that serum MMP-8 levels increase in colorectal cancer (CRC) and correlate with distant metastasis. However, short follow-up in our prospective cohort did not enable survival analyses at the time of the first publication.

**Methods:**

Preoperative serum MMP-8 levels were measured by immunofluorometric assay in 271 CRC patients and related to clinicopathological parameters, markers of systemic inflammation (modified Glasgow Prognostic Score, mGPS; serum levels of C-reactive protein (CRP), albumin and 13 cytokines), the density of six types of tumour-infiltrating immune cells and survival.

**Results:**

Increased MMP-8 levels associated with higher mGPS and higher serum levels of CRP and several cytokines, including IL-1ra, IL-7 and IL-8 (*p* < 0.001 for all). Serum MMP-8 negatively correlated with tumour-infiltrating mast cells (invasive margin: *p* = 0.005, tumour centre: *p* = 0.010). The patients with high-serum MMP-8 levels (>100 ng/mL) had poor cancer-specific survival, independent of tumour stage, grade, lymphatic invasion, patient age, BRAF VE1 immunohistochemistry, mismatch repair deficiency, Immunoscore and mGPS (multivariate HR 2.12, 95% CI 1.21–3.71, *p* = 0.009).

**Conclusions:**

High-serum MMP-8 levels are associated with systemic inflammation and adverse outcome in CRC.

## Introduction

Matrix metalloproteinase-8 (MMP-8) is an endopeptidase mainly produced by neutrophils, but it is also expressed at low levels by a variety of other inflammatory, epithelial and stromal cells.^1^ MMP-8 is released from intracellular granules of neutrophils, when neutrophils are activated by proinflammatory mediators or damage-associated molecular patterns (DAMPs).^2,3^ It cleaves collagens, some cell adhesion proteins, growth factors and cytokines (e.g. CXCL5, IL-8 and CXCL9).^1^ Studies with MMP8-deficient mice have demonstrated the essential role of MMP8 in neutrophil infiltration and function.^4,5^

Several studies have reported a potential protective role of MMP-8 in cancer development and progression,^5–7^ although also opposite reports exist.^8^ MMP-8-deficient male mice are susceptible to developing carcinogen-induced skin tumours and exhibit an altered inflammatory response induced by carcinogens.^5^ In breast cancer, high plasma levels of MMP-8 may have a protective effect against lymph node metastasis,^9^ and in oral tongue squamous cell carcinoma, high tumour MMP-8 expression is associated with improved cancer-specific survival (CSS).^10^ However, in ovarian cancer, tumour expression of MMP-8 has been shown to positively correlate with cancer progression^11^ and in hepatocellular carcinoma, high-serum levels of MMP-8 have been reported to associate with worse overall survival (OS).^8^

Colorectal cancer (CRC) is one of the most common malignancies and causes of cancer deaths in the western world.^12^ We have previously shown that serum MMP-8 levels increase in CRC and correlate with distant metastasis and weaker tumour inflammatory cell infiltrate, as assessed from the haematoxylin and eosin (H&E) slides.^13^ This led us to hypothesise that high-serum MMP-8 levels could be associated with adverse patient outcome. However, short follow-up in our prospective cohort did not allow for survival analyses at the time of the first publication. Moreover, more detailed analyses of serum MMP-8 levels in CRC in relation to different types of tumour-infiltrating immune cells and systemic inflammatory markers could improve our understanding of tumour-related inflammatory reactions.

Our previous study of serum MMP-8 levels in CRC relative to healthy controls included 116 patients with no preoperative oncological treatments (Cohort 1). For the present study, we extended the original cohort with 155 prospectively recruited CRC patients with no preoperative oncological treatments (Cohort 2). The primary aim was to assess the prognostic significance of serum MMP-8 levels in CRC patients. In addition, we aimed to analyse whether the earlier reported associations between serum MMP-8 levels and clinicopathological characteristics could be confirmed in an independent cohort. Moreover, to achieve a detailed picture of the relationships between serum MMP-8 levels and tumour-related inflammatory and immune reactions, we analysed the correlations between serum MMP-8 levels, six types of tumour-infiltrating immune cells, and serum levels of 13 cytokines, C-reactive protein (CRP) and albumin.

## Materials and methods

### Patients

This study protocol was introduced to all newly diagnosed CRC patients operated in Oulu University Hospital in 2006–2014, and the patients who signed an informed consent to participate were included. The study comprised two successive, prospectively recruited CRC cohorts; the patients in the original cohort (Cohort 1) were operated between 2006 and January 2010 (*n* = 149) and those in extension cohort (Cohort 2) between February 2010 and 2014 (*n* = 208).^13–15^ The patients with inadequate serum sample material for serum MMP-8 measurement were excluded (Cohort 1: *n* = 1; Cohort 2: *n* = 15). The patients signed a written informed consent to participate in the study, and the study design was approved by the Ethics Committee of Oulu University Hospital (58/2005, 184/2009). All the experiments were conducted in accordance with the Declaration of Helsinki. The details of age, gender, height, weight, medication and previous illnesses of the patients were acquired from clinical records and by a questionnaire. Survival data was collected from the clinical records and from Statistics Finland.^15,16^ CSS was defined as time from the operation to death from the same cancer, and OS was defined as time from the operation to death, irrespective of cause. Those who received preoperative radiotherapy/chemoradiotherapy (RT/CRT) (Cohort 1, *n* = 32; Cohort 2, *n* = 38) were excluded from analyses, as RT/CRT is a potential confounding factor affecting the inflammatory reaction around the tumour,^17^ so that the total number of patients included in this study was 271 (Cohort 1: *n* = 116, Cohort 2: *n* = 155). The REMARK guidelines were taken into account in the study design.^18^

### Blood analyses

Preoperative blood and serum samples of CRC patients were collected.^13,14^ The samples were stored at −70 °C until the analysis. Serum MMP-8 concentrations were determined with a time-resolved immunofluorometric assay (IFMA), as described earlier.^13,19^ Blood leukocyte counts, serum CRP levels and serum albumin levels were measured in the laboratory of Oulu University Hospital, and modified Glasgow Prognostic Score (mGPS) was calculated from CRP and albumin values.^14^ For Cohort 1, serum concentrations of 27 cytokines were measured by Bio-Plex Pro Human pre-manufactured 27-Plex Cytokine Panel (Bio-Rad, Hercules, CA, USA).^14^ As expanded on earlier, 14 cytokines had many values below or above the assay detection limits, and 13 cytokines with less than four values outside the assay working range were included in this study.^14^ All the assays were performed blinded to the clinical and pathological data.

### Histopathological analysis of the tumours, immunohistochemistry and immune cell counting

The staging of the tumours was conducted according to TNM6 (Cohort 1) or TNM7 (Cohort 2), and the grading according to the World Health Organization (WHO) criteria (both cohorts). Lymphatic invasion and blood vessel invasion were evaluated from the H&E stained sections. Lymphatic invasion was categorised positive in cases that had tumour cells present in vessels with an endothelial lining but lacking a muscular wall, while blood vessel invasion was defined as tumour cells in vessels with a thick muscular wall or in vessels containing red blood cells.^20^

For both cohorts, tissue microarrays (TMAs) with one to four cores of 3.0 mm diameter (Cohort 1, median 3; Cohort 2, median 4), depending on the size of the tumour, including both the invasive margin (IM) and the centre of the tumour (CT), were constructed.^21,22^ Immunohistochemistry was performed on 3.5 µm sections cut from the TMA paraffin blocks for six immune cell markers (CD3, CD8, FoxP3, CD68, Mast cell tryptase and Neutrophil elastase; Table S1).^21^ For immune cell counting, images were captured from the CT, and the IM and the cell densities were counted using a computer-assisted cell counting method^23^ that utilises ImageJ, a freeware image analysis software.^24^ Intraepithelial (CT-IEL) immune cells (CD3, CD8) were counted manually from the captured images due to the inadequacy of the automatic cell counting to segregate the intraepithelial cells from those in the stroma.^21^ To determine Immunoscore,^25^ CD3^+^ and CD8^+^ T-cell densities at the IM and in the CT were recoded into two-tiered categorical variables (low = 0 vs. high = 1) using the median as the cut-off point. The Immunoscore was then defined as the sum of these four variables (ranging from 0 to 4).

Mismatch repair (MMR) enzyme status was analysed utilising MLH1, MSH2, MSH6 and PMS2 immunohistochemistry.^13,15,26^ BRAF V600E-specific VE1 immunohistochemistry was conducted with Ventana Bench-Mark XT immunostainer (Ventana Medical Systems, Tucson, AZ) to evaluate *BRAF* mutation status of both cohorts.^27^ Our earlier study has indicated that the method had a sensitivity of 100% and a specificity of 99.3% in detecting *BRAF* V600E mutation.^27^ All analyses were blinded to the clinical data and serum data.

### Statistical analyses

The statistical analyses were carried out using IBM SPSS Statistics for Windows version 22.0 (IBM Corporation, Armonk, NY, USA). Normally distributed continuous variables are presented as mean (standard deviation, SD) and other continuous variables are presented as median (interquartile range, IQR). Pearson correlation coefficients (*r*) were used in examining correlations between two continuous variables. A logarithmic transformation was applied to variables with positive skewness. Multiple linear regression was conducted to adjust the correlations for additional parameters. Statistical significances of the associations between continuous and categorical variables were analysed by Mann–Whitney *U* test or Kruskal–Wallis test, and statistical significances of the associations between two categorical variables were analysed by *χ*^2^ test or Fisher’s exact test. Receiver operating characteristics (ROC) analysis was used to determine the optimal cut-off score for serum MMP-8 level in discriminating survivors from non-survivors and to compare the discrimination ability of different parameters (area under the curve, AUC). Kaplan–Meier method, log-rank test and Cox regression analysis were used in the survival analyses. In multivariate models, the cases with one or more missing values were excluded. The 2D visualisation of the relationships between serum MMP-8 levels and other systemic inflammatory markers was created with Cytoscape software platform,^28^ utilising the Prefuse force directed algorithm weighted by the statistical significances of the correlations between individual variables. A two-tailed *p* < 0.05 was considered statistically significant.

## Results

### Serum MMP-8 levels in relation to basic clinicopathological parameters

The study included two successive CRC patient cohorts, and Table 1 shows the characteristics of Cohort 1, Cohort 2 and the combined cohort. The median serum MMP-8 level was 56.8 ng/mL in Cohort 1, 68.4 ng/mL in Cohort 2 and 64.3 ng/mL in the combined cohort.

**Table 1:** Characteristics of colorectal cancer patient cohorts

	Cohort 1 (*n* = 116)	Cohort 2 (*n* = 155)	Combined cohort (*n* = 271)
Age, mean (SD)	67.6 (11.2)	70.8 (11.8)	69.5 (11.6)
Sex			
Male	58 (50%)	80 (51.6%)	138 (50.9%)
Female	58 (50%)	75 (48.4%)	133 (49.1%)
Tumour location			
Proximal colon	48 (41.4%)	67 (43.2%)	115 (42.4%)
Distal colon	28 (24.1%)	44 (28.4%)	72 (26.6%)
Rectum	40 (34.5%)	44 (28.4%)	84 (31.0%)
WHO grade			
Grade 1	16 (13.9%)	51 (32.9%)	67 (24.8%)
Grade 2	86 (74.8%)	85 (54.8%)	171 (63.3%)
Grade 3	13 (11.3%)	19 (12.3%)	32 (11.9%)
TNM stage			
Stage I	19 (16.7%)	40 (25.8%)	59 (21.9%)
Stage II	45 (39.5%)	44 28.4%)	89 (33.0%)
Stage III	32 (28.1%)	51 (32.9%)	81 (30.0%)
Stage IV	18 (15.8%)	20 (12.9%)	41 (15.2%)
Mismatch repair status			
Deficient	11 (9.6%)	27 (17.4%)	38 (14.1%)
Proficient	104 (90.4%)	128 (82.6%)	232 (85.9%)
mGPS			
0	91 (78.4%)	118 (76.6%)	209 (77.4%)
1	21 (18.1%)	33 (21.4%)	54 (20.0%)
2	4 (3.4%)	3 (1.9%)	7 (2.6%)
Serum matrix metalloproteinase-8, ng/mL, median (IQR)	56.8 (20.5–124.6)	68.4 (39.2–108.0)	64.3 (27.9–118.4)
Serum CRP, mg/L, median (IQR)	2.10 (0.81–7.75)	3.39 (0.86–9.73)	2.8 (0.81–9.10)
Serum albumin, g/L, median (IQR)	43.0 (40.25–46.0)	43.0 (40.0–45.0)	43.0 (40.0–45.0)

*CRP* C-reactive protein, *IQR* interquartile range, *SD* standard deviation

Our earlier study,^13^ utilising Cohort 1, suggested that increased serum MMP-8 are associated with higher TNM stage, higher T-class and the presence of distant metastasis. In the current study, the analyses in Cohort 2 confirmed these result (Table S2). Since these two cohorts represent a continuous series of patients, we combined them for subsequent analyses to increase the statistical power. The analyses of clinicopathological correlations in the combined cohort are displayed in Table S3. In addition to higher TNM stage (*p* < 0.001), T-class (*p* = 0.001) and M-class (*p* < 0.001) increased serum MMP-8 as a continuous variable was associated with lymphatic invasion (*p* < 0.001), blood vessel invasion (*p* = 0.001), low-grade peritumoural inflammatory infiltrate (*p* = 0.014), low Immunoscore (*p* = 0.050) and positive BRAF VE1 immunohistochemistry indicating *BRAF* V600E mutation (*p* = 0.033). As a categorical variable (Table S4), high-serum MMP-8 ( > 100 ng/mL) was associated with higher TNM stage (*p* < 0.001), T-class (*p* = 0.001) and M-class (*p* < 0.001), lymphatic invasion (*p* = 0.001), blood vessel invasion (*p* = 0.004), positive BRAF VE1 immunohistochemistry (*p* = 0.012) and MMR deficiency (*p* = 0.050).

### Correlation of serum MMP-8 with systemic inflammation markers, cytokines and tumour-infiltrating immune cells

**Fig. 1:**
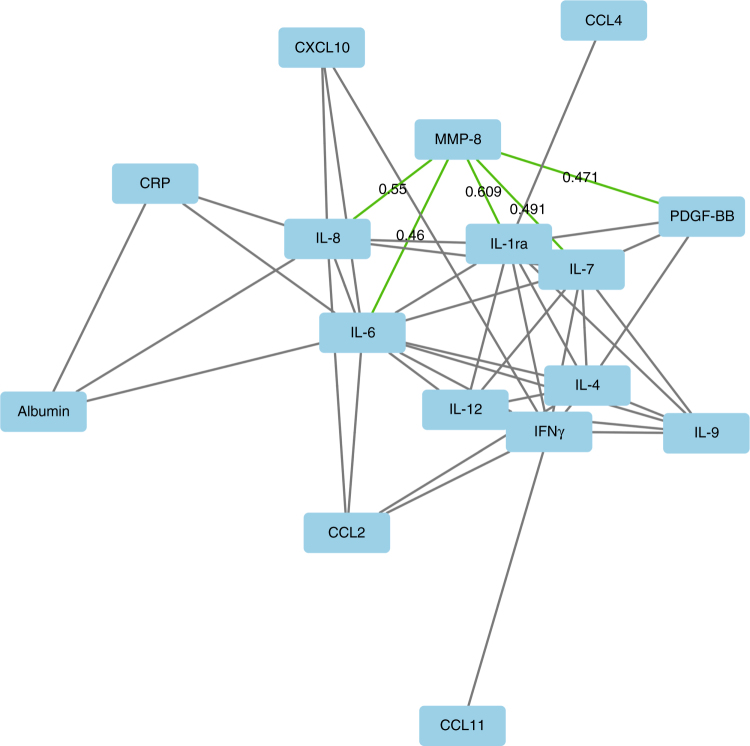
2D visualisation of the relationships between serum MMP-8, serum CRP, serum albumin and serum cytokine levels in Cohort 1. Individual variables are represented by nodes and their associations are represented by edges (connecting lines). Only the associations with Pearson *r* > 0.400 or Pearson *r* < −0.300 are shown, and the edge length illustrates the significance of the association. Grey edges indicate associations between cytokines, CRP and albumin, while the correlations between MMP-8 and cytokines are represented by green (positive correlation) edges, with the label indicating corresponding Pearson *r* for the correlation. *CCL* Chemokine (C–C motif) ligand, *CRP* C-reactive protein, *CXCL* Chemokine (C–X–C motif) ligand, *IFN* interferon, *IL* interleukin, *MMP* matrix metalloproteinase, *PDGF* platelet-derived growth factor

We evaluated the correlations between serum MMP-8 and systemic inflammatory markers (Table 2, Fig. 1, Tables S3-S5). Serum MMP-8 had strong positive correlations with mGPS, serum CRP, blood neutrophil count and blood neutrophil/lymphocyte ratio (NLR) (*p* < 0.001 for all). Serum levels of 13 cytokines were measured in Cohort 1, and serum MMP-8 levels positively correlated with several of them; the strongest correlations were between serum MMP-8 and serum IL-1ra, serum IL-7 and serum IL-8 (*p* < 0.001 for all).

**Table 2:** Correlations between serum MMP-8 levels, markers of systemic inflammation and the density of tumour-infiltrating inflammatory cells

	Unadjusted		Adjusted	
	Pearson *r*	*p* Value	*β*	*p* Value
Systemic inflammatory markers				
Serum C-reactive protein	0.324	5.9E–8	0.220	2.3E–4
Serum albumin	−0.203	0.001	−0.130	0.026
Blood neutrophil/lymphocyte ratio	0.436	6.8E–14	0.379	3.0E–11
Blood neutrophil count	0.467	5.9E–16	0.399	1.9E–12
Blood lymphocyte count	−0.099	0.106	−0.080	0.167
Tumour-infiltrating immune cells				
CD3 IM	−0.162	0.008	−0.045	0.461
CD3 CT	−0.067	0.268	0.020	0.745
CD3 IEL	−0.068	0.267	0.024	0.696
CD8 IM	−0.073	0.233	0.030	0.616
CD8 CT	−0.067	0.272	0.014	0.818
CD8 IEL	−0.024	0.701	0.060	0.327
FoxP3 IM	−0.198	0.001	−0.071	0.267
FoxP3 CT	−0.187	0.002	−0.060	0.358
CD68 IM	−0.075	0.221	−0.001	0.980
CD68 CT	0.009	0.884	0.071	0.223
Mast cell tryptase IM	−0.245	4.6E–5	−0.167	0.005
Mast cell tryptase CT	−0.179	0.003	−0.149	0.010
Neutrophil elastase IM	−0.099	0.109	−0.009	0.794
Neutrophil elastase CT	−0.025	0.688	0.008	0.894

The correlations were adjusted for tumour stage variables (T1–2 vs. T3–4; N0 vs. N1–2; M0 vs. M1), patient age and patient gender by multiple linear regression.

*CT* centre of tumour, *IEL* intraepithelial, *IM* invasive margin

We analysed the correlations between serum MMP-8 levels and the densities of six types of tumour-infiltrating immune cells (Table 2). Serum MMP-8 showed negative correlation with tumour-infiltrating mast cells (IM: tumour stage, patient age and patient gender adjusted *p* = 0.005; tumour centre: tumour stage, patient age and patient gender adjusted *p* = 0.010). However, the densities of tumour infiltrating CD3^+^, CD8^+^, FoxP3^+^ T cells, CD68^+^ macrophages and neutrophils did not significantly correlate with serum MMP-8 levels, when the correlations were adjusted for tumour stage, patient age and patient gender.

### Survival analyses

**Fig. 2:**
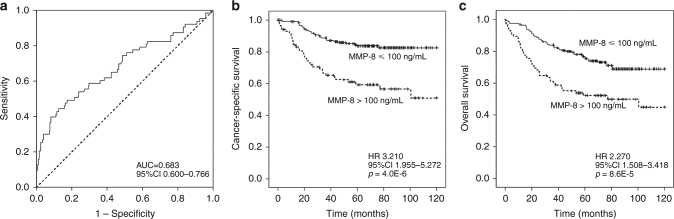
Survival analysis. **a** Receiver operating characteristics (ROC) analysis for serum MMP-8 in separating survivors from non-survivors. **b** Kaplan–Meier curve for serum MMP-8 and CSS. **c** Kaplan–Meier curve for serum MMP-8 and OS

The primary aim of this study was to investigate the prognostic significance of serum MMP-8 levels, and 120-month survival analysis was performed in the combined cohort (Fig. 2, Table 3). ROC analysis indicated that a wide range of serum MMP-8 levels was capable of discriminating survivors from non-survivors (AUC = 0.683, 95% CI = 0.600−0.766), and the survival was decreased in higher serum MMP-8 levels (Table S6). A cut-off point of 100 ng/mL for serum MMP-8, was chosen because it had a short distance to the coordinate (0,1) in ROC curve and it was considered easily reproducible. The AUC of serum MMP was higher than that of systemic inflammatory markers NLR (AUC = 0.593), blood neutrophil count (AUC = 0.599) and serum CRP (AUC = 0.611). Of other studied prognostic parameters, TNM stage (AUC = 0.858), N-class (AUC = 0.789), M-class (AUC = 0.732), lymphatic invasion (AUC = 0.720) and Immunoscore (AUC = 0.722) had higher AUCs than serum MMP-8, whereas the AUCs of T-class (AUC = 0.614), grade (AUC = 0.606), blood vessel invasion (AUC = 0.662), MMR status (AUC = 0.581) and BRAF VE1 immunohistochemistry (AUC = 0.508) were lower than that of serum MMP-8. Serum MMP-8 in combination with Immunoscore (AUC = 0.744), serum CRP (AUC = 0.686) or lymphatic invasion (AUC = 0.756) showed higher AUCs than separate variables (Table S6). Kaplan–Meier curves indicated that increased serum MMP-8 (>100 ng/mL) was associated with worse CSS (*p* < 0.001) and OS (*p* < 0.001) (Fig. 2). In multivariate analysis, MMP-8 was independent prognostic factor for CSS (HR 2.12, 95% CI 1.21–3.71, *p* = 0.009) (Table 3). The association of MMP-8 and adverse CSS was also evident when the analysis was restricted to 60 months (Table S7).

**Table 3:** Multivariate analysis of 120-month cancer-specific survival (CSS) and overall survival (OS) of CRC patients in the combined cohort

	CSS			OS		
	HR	95% CI	*p* value	HR	95% CI	*p* value
Age (<65 vs. ≥65)	1.69	0.93–3.06	0.083	2.29	1.36–3.87	0.002
Tumour invasion (T1–T2 vs. T3–T4)	0.64	0.31–1.30	0.217	0.71	0.41–1.23	0.220
Nodal metastases (N0 vs. N1–N2)	3.98	1.81–8–74	0.001	2.23	1.28–3.88	0.005
Distant metastases (M0 vs. M1)	6.09	3.16–11.7	6.8E–8	3.83	2.19–6.72	2.8E–6
Lymphatic invasion (No vs. Yes)	1.36	0.62–2.96	0.443	1.12	0.64–1.97	0.693
Grade (1–2 vs. 3)	2.10	1.30–3.39	0.002	1.83	1.26–2.65	0.001
mGPS (0 vs. 1–2)	1.03	0.55–3.39	0.931	1.37	0.82–2.29	0.227
BRAF VE1 immunohistochemistry (negative vs. positive)	2.65	0.96–7.28	0.059	1.34	0.59–3.07	0.488
MMR status (proficient vs. deficient)	0.16	0.02–1.30	0.087	0.83	0.35–1.94	0.667
Immunoscore (0–1 vs. 2–4)	0.47	0.26–0.87	0.016	0.50	0.31–0.81	0.004
Serum MMP-8 (≤100 ng/mL vs. >100 ng/mL)	2.12	1.21–3.71	0.009	1.45	0.91–2.30	0.118

CSS: *n* = 264; median follow-up time 64.3 months (IQR 36.5–85.5); 60 (22.2%) events; 7 (2.6%) cases excluded from the analysis because of missing values.

OS: *n* = 266; median follow-up time 64.3 months (IQR 36.5–85.5); 90 (33.2%) events; 5 (1.8%) cases excluded from the analysis because of missing values.

*CI* confidence interval, *HR* hazard ratio

## Discussion

Matrix degradation and the regulation of leukocyte recruitment and accumulation are important events in cancer initiation and progression.^29^ MMP-8 has been reported to harbour both pro- and anti-inflammatory functions, as well as pro- and anti-tumour functions.^1^ However, the prognostic significance of serum MMP-8 in CRC, as well as its associations with systemic inflammatory markers in CRC, have been unknown.

The main findings of the present study indicate that high-serum MMP-8 levels are associated with adverse CSS in CRC independent of other prognostic parameters, including TNM stage, grade, lymphatic invasion, BRAF VE1 immunohistochemistry, MMR deficiency, Immunoscore and mGPS. Moreover, strong positive correlations were detected between serum MMP-8 and serum levels of CRP and several cytokines, including IL-1ra, IL-7 and IL-8, indicating that serum MMP-8 levels are associated with systemic inflammation in CRC.

In CRC, systemic inflammatory response has been associated with decreased survival,^30^ whereas a dense intratumoural immune cell infiltrate is a marker of better prognosis.^25,31,32^ mGPS and NLR reflect systemic inflammation, and several studies have indicated that they are potential prognostic parameters in CRC.^30,33^ Our analyses show that serum MMP-8 levels are closely related to systemic inflammatory markers, suggesting a physiological link between MMP-8 and systemic inflammation. Systemic inflammatory response after tissue trauma may be elicited as DAMPs are released to the circulation, potentially leading to neutrophil activation and MMP-8 secretion.^3^ In CRC, the mechanism eliciting systemic inflammatory response is not yet known, but tumour necrosis has been shown to associate with markers of systemic inflammation.^34^ Interestingly, higher serum MMP-8 levels are also associated with tumour necrosis.^13^ Also supporting the link between MMP-8 and systemic inflammation, earlier studies have shown that circulating MMP-8 levels positively correlate with CRP levels in acute coronary syndrome,^35^ and increased MMP-8 levels have been reported in several chronic inflammatory conditions like sepsis,^36^ rheumatoid arthritis^37^ and periodontal disease.^38^

To generate a more detailed overview of the relationships between serum MMP-8 and circulating inflammatory mediators, we analysed the correlations between serum MMP-8 and serum levels of 13 cytokines. Our results displayed that serum MMP-8 levels positively correlated with the levels of 12 of these cytokines, forming a cluster with IL-1ra, IL-6, IL-7 and IL-8 in the centre of the 2D illustration (Fig. 1). IL-1ra is a specific inhibitor of the activity of both IL-1α and IL-1β, thus exerting anti-inflammatory function.^39^ Therefore, the functions of MMP-8 and IL-1ra could complement each other, since also MMP-8 can downregulate inflammatory reactions by cleaving pro-inflammatory cytokines or chemokines.^1^ IL-7 regulates T-cell homoeostasis, but its role in CRC is unclear.^40^ IL-6 is a major pro-inflammatory cytokine, capable of activating signalling pathways that promote tumour progression and regulate the secretion of other cytokines.^41^ Interestingly, MMP-8 has been shown to induce the expression of IL-6 and IL-8 in breast cancer cells,^42^ but it is not known, whether this function takes place in CRC cells in vivo. IL-8 is an important chemoattractant for neutrophils, and we hypothesise that the strong association between serum IL-8 and MMP-8 may be related to this role, since neutrophils are considered major contributors of MMP-8 production.^1^ Thus, serum levels of MMP-8 are closely related to several important circulating inflammatory mediators.

In this study, we did not observe correlation between the density of tumour-infiltrating neutrophils and serum MMP-8 levels, although our earlier study showed MMP-8 expression in tumour-infiltrating neutrophils in CRC.^13^ Instead, there is strong correlation between circulating neutrophil counts and serum MMP-8 levels.^13^ This suggests that the neutrophils in circulation may be an important source of serum MMP-8. Tumour-infiltrating neutrophils show plasticity, and based on murine models, separate anti-tumour N1 and pro-tumour N2 neutrophil subsets have been suggested.^43^ However, the definitions and reliable markers for different neutrophil subsets in human patients still require further research,^44^ and subsequent studies concentrating on diverse tumour-infiltrating neutrophil subsets could enlighten the potential role of MMP-8 in these cells. Of the studied tumour-infiltrating inflammatory cells, serum MMP-8 levels only showed negative correlation with mast cells, when adjusted for tumour stage, patient age, and patient gender, but not with tumour-infiltrating T cells, which are considered more important in anti-tumour immunity.^29^ There is inadequate data on the role of MMP-8 in regulating mast cell function, requiring further investigation.

Currently, TNM stage is the main prognostic and predictive parameter for CRC, and lymphatic and vascular invasion are among the most widely utilised supplementary markers.^45,46^ However, each tumour and patient is unique,^47^ and additional parameters could help to better stratify the patients to receive optimal treatments. The present study indicates that increased serum MMP-8 levels are associated with adverse CSS and OS in CRC. In multivariate models, increased serum MMP-8 associated with worse CSS independent of other clinicopathological variables, including age, TNM stage, grade, lymphatic invasion, BRAF VE1 immunohistochemistry, MMR deficiency, Immunoscore and mGPS. This result suggests that serum MMP-8 could be a relevant additional prognostic parameter in CRC. Notably, the prognostic significance of serum MMP-8 in the multivariate Cox regression model of CSS was higher than that of lymphatic invasion or mGPS, both of which have previously shown to possess independent prognostic value in several studies.^30,48^ We tested combinatory prognostic variables, and our analyses indicated that serum MMP-8 could be combined with a variety of variables to improve the prognostic power. For example, the combination of Immunoscore and serum MMP-8, reflecting both anti-tumour immune response and systemic inflammation, achieved good discrimination ability in ROC analysis and both parameters significantly contributed to the multivariate Cox regression model of CSS. However, our study was based on unselected CRC patient material of stages I–IV and further studies are required to assess the prognostic performance of serum MMP-8 in more strictly defined and therapeutically relevant subgroups such as stage II patients.

Some limitations need to be considered in the interpretation of the results. First, previous studies have indicated that serum samples may have higher MMP levels than plasma samples due to molecules released during the clotting process.^3,49^ For our study, plasma samples were not available. However, serum MMP-8 levels have been shown to have significant positive correlation with plasma MMP-8 levels in the IFMA assay that was used in this study.^50^ Nevertheless, further studies are required to compare the prognostic significance of serum and plasma MMP-8 levels in CRC. Second, the study setup was not designed for the analyses of a potential predictive role of circulating MMP-8 levels in specific patient subgroups, such as stage II or stage III patients or patients who had received neoadjuvant treatment, which is an important subject for further investigations. The strength of the study is that it was prospectively recruited with a well characterised study population with consistent and extensive histopathological analysis including additional prognostic parameters such as lymphatic and blood vessel invasion. An assemblage of systemic inflammatory markers and tumour-infiltrating immune cells were analysed, which enabled a more in-depth view of the relationships between serum MMP-8 levels and tumour associated inflammatory reactions than the analyses of a single marker.

In conclusion, serum MMP-8 levels positively correlate with systemic inflammatory markers, including mGPS and serum levels of CRP, IL-1ra, IL-7 and IL-8. Serum MMP-8 is associated with adverse CSS in CRC, independent of tumour stage, grade, lymphatic invasion, BRAF VE1 immunohistochemistry, MMR deficiency, Immunoscore and mGPS. Further studies are warranted to confirm the prognostic value of serum MMP-8 in CRC in specific patient subgroups and to assess the predictive value of serum MMP-8 in CRC.

## Electronic supplementary material

Supplementary online material

## Data Availability

The data sets generated and analysed during the current study are available from the corresponding author on reasonable request.
