# Supplementary material for: High-serum MMP-8 levels are associated with decreased survival and systemic inflammation in colorectal cancer
Source: Br J Cancer. 2018 May 29;119(2):213–9. doi: 10.1038/s41416-018-0136-4 (PMC6048114; doi:10.1038/s41416-018-0136-4)
Supplement: Supplementary file 1 — Supplementary online material [file 41416_2018_136_MOESM1_ESM.pdf]

Supplementary online material

High serum MMP-8 levels are associated with decreased survival and systemic inflammation in colorectal cancer

Päivi Sirniö[1,2], Anne Tuomisto[1,2], Taina Tervahartiala[3], Timo Sorsa[3], Kai Klintrup[2,4], Toni Karhu[5], Karl-Heinz Herzig[2,5,6], Jyrki Mäkelä[2,4], Tuomo J. Karttunen[1,2], Tuula Salo[1,2,3,7], Markus J. Mäkinen[1,2], Juha P. Väyrynen[1,2]

[1]Cancer and Translational Medicine Research Unit, University of Oulu, POB 5000, 90014 Oulu, Finland
[2]Oulu University Hospital and Medical Research Center Oulu, POB 21, 90029 Oulu, Finland
[3]Department of Oral and Maxillofacial Diseases, University of Helsinki, Haartmaninkatu 8, POB 63, 00014, Helsinki Finland
[4]Research Unit of Surgery, Anesthesia and Intensive Care, University of Oulu, POB 5000, 90014 Oulu, Finland
[5]Research Unit of Biomedicine and Biocenter Oulu, Department of Physiology, University of Oulu, POB 5000, 90014 Oulu, Finland
[6]Department of Gastroenterology and Metabolism, Poznan University of Medical Sciences, ul. Szpitalna 27/33, 60-572, Poznan, Poland
[7]Helsinki University Hospital, 00014 Helsinki, Finland

Table S1. Antibodies and protocols used in immunohistochemistry

Cell type	Antigen	Antigen retrieval	Antibody type	Manufacturer	Clone	Code	Dilution	Incubation	Antibody visualization
T cells	CD3	Tris-EDTA pH9*	mouse monoclonal	Novocastra	PS1	NCL-CD3-PS1	1:50	30 min	EnVision
Cytotoxic T cells	CD8	Tris-EDTA pH9*	mouse monoclonal	Novocastra	4B11	NCL-CD8-4B11	1:200	30 min	EnVision
Regulatory T cells	FoxP3	Tris-EDTA pH9*	mouse monoclonal	Abcam	236A/E7	ab20034	1:100	30 min	EnVision
Macrophages	CD68	Tris-EDTA pH9*	mouse monoclonal	DAKO	PG-M1	M0876	1:100	30 min	EnVision
Neutrophils	Neutrophil elastase	Tris-EDTA pH9*	mouse monoclonal	DAKO	NP57	M0752	1:200	30 min	EnVision
Mast cells	Mast cell tryptase	Tris-EDTA pH9*	mouse monoclonal	DAKO	AA1	M7052	1:2000	30 min	EnVision
MLH1^{+} cells	MLH1	Tris-EDTA pH9*	mouse monoclonal	Novocastra	ES05	NCL-L-MLH1	1:100	90 min	EnVision
MSH2^{+} cells	MSH2	Tris-EDTA pH9*	mouse monoclonal	BD-Pharmingen	G219-1129	556349	1:200	60 min	EnVision
MSH6^{+} cells	MSH6	Tris-EDTA pH9*	mouse monoclonal	BD-Pharmingen	44/MSH6	610919	1:150	90min	EnVision
PMS2^{+} cells	PMS2	Tris-EDTA pH9*	mouse monoclonal	BD-Pharmingen	A16-4	556415	1:100	90 min	EnVision
BRAF V600E mutated cells	BRAF V600E	Ventana CC1	mouse monoclonal	Spring Bioscience	VE1	E19292	1:2000	32 min	Optiview

*In a microwave oven at 800W for 2 min and at 150W for 15 min.

Table S2. Serum MMP-8 levels in relation to clinical and pathological characteristics of CRCs in Cohort 2

	MMP-8, median (IQR)	p value
**Age**		
< 65 (n=44)	55.8 (33.4-83.6)	0.085
≥ 65 (n=111)	74.0 (42.8-121.2)	
**Sex**		
Male (n=80)	68.4 (37.8-106.5)	0.427
Female (n=75)	68.4 (45.2-118.4)	
**BMI**		
<25 (n=47)	60.8 (39.2-100.8)	0.451
25-30 (n=61)	68.4 (37.4-105.8)	
>30 (n=41)	74.4 (45.8-124.2)	
**Tumor location**		
Proximal colon (n=67)	76.4 (45.6-120.0)	0.414
Distal colon (n=44)	58.2 (40.8-87.5)	
Rectum (n=44)	64.6 (27.6-105.8)	
**WHO grade**		
Grade 1 (n=51)	53.2 (34.0-120.0)	0.341
Grade 2 (n=85)	68.4 (43.0-101.6)	
Grade 3 (n=19)	94.0 (56.4-121.2)	
**TNM stage**		
Stage I (n=40)	71.2 (37.6-98.6)	0.005
Stage II (n=44)	63.4 (34.9-107.8)	
Stage III (n=51)	63.6 (32.8-100.4)	
Stage IV (n=20)	122.8 (61.9-258.3)	
**Depth of invasion**		
T1 (n=11)	51.6 (37.2-158.4)	0.015
T2 (n=40)	75.8 (41.2-101.0)	
T3 (n=89)	60.8 (38.0-99.6)	
T4 (n=15)	122.4 (65.2-416.0)	
**Nodal metastases**		
N0 (n=85)	68.4 (37.4-105.8)	0.227
N1 (n=48)	66.4 (34.3-143.1)	
N2 (n=22)	75.6 (52.6-120.6)	
**Distant metastases**		
M0 (n=135)	64.0 (37.2-100.4)	3.6E-4
M1 (n=20)	122.8 (61.9-258.3)	
**Lymphatic invasion**		
No (n=79)	66.0 (38.4-93.2)	0.111
Yes (n=76)	71.8 (44.4-125.1)	
**Blood vessel invasion**		
No (n=131)	66.0 (38.4-104.4)	0.049
Yes (n=24)	84.8 (56.5-208.6)	
**Peritumoral inflammatory cell infiltrate (Klintrup-Mäkinen score)**		
Low-grade (n=60)	72.2 (47.3-124.5)	0.106
High-grade (n=95)	66.0 (37.2-104.4)	
**Immunoscore**		
0-1 (n=66)	72.2 (46.8-123.3)	0.133
2-4 (n=89)	66.0 (34.8-107.2)	
**BRAF VE1 immunohistochemistry**		
Negative (n=138)	63.8 (38.2-101.6)	0.021
Positive (n=17)	107.2 (67.0-140.6)	
**MMR enzyme status**		
MMR-deficient (n=27)	82.0 (53.2-128.4)	0.174
MMR-proficient (n=128)	64.6 (38.8-101.4)	
**Modified Glasgow Prognostic Score**		
0 (n=118)	62.2 (37.0-100.5)	0.014
1 (n=33)	76.4 (46.6-167.2)	
2 (n=3)	356.0 (215.6-398.8)	

Abbreviations: IQR: interquartile range, MMR: mismatch repair. P values are for Mann-Whitney or Kruskal-Wallis test.

Table S3. Serum MMP-8 levels in relation to clinical and pathological characteristics of CRCs in the combined cohort

	MMP-8, median (IQR)	p value
**Age**		
< 65 (n=87)	53.2 (27.9-91.2)	0.138
≥ 65 (n=184)	70.6 (28.9-123.4)	
**Sex**		
Male (n=138)	60.8 (27.2-108.6)	0.290
Female (n=133)	68.8 (30.6-123.6)	
**BMI**		
<25 (n=94)	68.5 (32.0-126.6)	0.390
25-30 (n=106)	63.0 (22.9-108.6)	
>30 (n=63)	66.0 (28.4-123.6)	
**Tumor location**		
Proximal colon (n=115)	72.3 (32.8-122.4)	0.406
Distal colon (n=72)	61.4 (36.6-116.0)	
Rectum (n=84)	54.8 (24.9-118.4)	
**WHO grade**		
Grade 1 (n=67)	47.6 (22.8-107.2)	0.153
Grade 2 (n=171)	68.4 (31.3-121.1)	
Grade 3 (n=32)	83.0 (34.3-145.2)	
**TNM stage**		
Stage I (n=59)	51.6 (30.0-88.0)	6.8E-7
Stage II (n=89)	63.5 (23.3-110.3)	
Stage III (n=81)	56.4 (23.9-101.6)	
Stage IV (n=41)	123.2 (69.0-252.0)	
**Depth of invasion**		
T1 (n=16)	50.8 (19.9-110.7)	0.001
T2 (n=59)	54.0 (27.9-91.2)	
T3 (n=171)	63.6 (27.2-117.9)	
T4 (n=24)	158.0 (66.5-382.2)	
**Nodal metastases**		
N0 (n=153)	62.6 (27.6-107.6)	0.094
N1 (n=75)	64.0 (26.8-149.2)	
N2 (n=41)	72.8 (46.4-136.1)	
**Distant metastases**		
M0 (n=230)	56.6 (24.7-102.3)	2.8E-8
M1 (n=41)	123.2 (69.0-252.0)	
**Lymphatic invasion**		
No (n=144)	50.4 (24.0-90.9)	3.1E-4
Yes (n=124)	76.8 (44.4-149.1)	
**Blood vessel invasion**		
No (n=221)	60.8 (26.2-107.2)	0.001
Yes (n=47)	94.0 (56.4-166.8)	
**Peritumoral inflammatory cell infiltrate (Klintrup-Mäkinen score)**		
Low-grade (n=125)	73.9 (36.5-134.5)	0.014
High-grade (n=146)	56.1 (27.1-105.5)	
**Immunoscore**		
0-1 (n=113)	72.8 (40.1-132.8)	0.050
2-4 (n=158)	59.1 (26.3-108.3)	
**BRAF VE1 immunohistochemistry**		
Negative (n=242)	63.0 (27.2-110.3)	0.033
Positive (n=29)	100.4 (55.3-140.6)	
**MMR enzyme status**		0.124
MMR-deficient (n=38)	78.6 (49.5-125.8)	
MMR-proficient (n=232)	63.5 (27.2-115.2)	
**Modified Glasgow Prognostic Score**		
0 (n=209)	59.2 (24.5-100.6)	1.0E-6
1 (n=54)	81.8 (50.0-194.7)	
2 (n=7)	280.9 (167.8-356.0)	

Abbreviations: IQR: interquartile range, MMR: mismatch repair. P values are for Mann-Whitney or Kruskal-Wallis test.

Table S4. Serum MMP-8 levels as two-tiered categorical variable in relation to clinical and pathological characteristics of CRCs in the combined cohort

	Serum MMP-8		
	**Low** (≤100 ng/mL)	**High** (>100 ng/mL)	**p value**
**Age**			
< 65 (n=87)	66 (75.9%)	21 (24.1%)	0.078
≥ 65 (n=184)	120 (65.2%)	64 (34.8%)	
**Sex**			
Male (n=138)	98 (71.0%)	40 (29.0%)	0.390
Female (n=133)	88 (66.2%)	45 (33.8%)	
**BMI**			
<25 (n=94)	63 (67.0%)	31 (33.0%)	0.802
25-30 (n=106)	75 (70.8%)	31 (29.2%)	
>30 (n=63)	42 (66.7%)	21 (33.3%)	
**Tumor location**			
Proximal colon (n=115)	75 (65.2%)	40 (34.8%)	0.561
Distal colon (n=72)	52 (72.2%)	20 (27.8%)	
Rectum (n=84)	59 (70.2%)	25 (29.8%)	
**WHO grade**			
Grade 1 (n=67)	49 (73.1%)	18 (26.9%)	0.384
Grade 2 (n=171)	118 (69.0%)	53 (31.0%)	
Grade 3 (n=32)	19 (59.4%)	13 (40.6%)	
**TNM stage**			
Stage I (n=59)	48 (81.4%)	11 (18.6%)	6.3E-5
Stage II (n=89)	63 (70.8 %)	26 (29.2%)	
Stage III (n=81)	59 (72.8%)	22 (27.2%)	
Stage IV (n=41)	16 (39.0%)	25 (61.0%)	
**Depth of invasion**			
T1 (n=16)	12 (75.0%)	4 (25.0%)	3.1E-4
T2 (n=59)	48 (81.4%)	11 (18.6%)	
T3 (n=171)	118 (69.0%)	53 (31.0%)	
T4 (n=24)	8 (33.3%)	16 (66.7%)	
**Nodal metastases**			
N0 (n=153)	112 (73.2%)	41 (28.6%)	0.169
N1 (n=75)	50 (66.7%)	25 (33.3%)	
N2 (n=41)	24 (58.5%)	17 (41.5%)	
**Distant metastases**			
M0 (n=230)	170 (73.9%)	60 (26.1%)	9.0E-6
M1 (n=41)	16 (39.0%)	25 (61.0%)	
**Lymphatic invasion**			
No (n=144)	112 (77.8%)	32 (22.2%)	0.001
Yes (n=124)	72 (58.1%)	52 (41.9%)	
**Blood vessel invasion**			
No (n=221)	160 (72.4%)	61 (27.6%)	0.004
Yes (n=47)	24 (51.1%)	23 (48.9%)	
**Peritumoral inflammatory cell infiltrate (Klintrup-Mäkinen score)**			
Low-grade (n=125)	80 (64.0%)	45 (36.0%)	0.128
High-grade (n=146)	106 (72.6%)	40 (27.4%)	
**Immunoscore**			
0-1 (n=113)	73 (64.6%)	40 (35.4%)	0.235
2-4 (n=158)	113 (71.5%)	45 (28.5%)	
**BRAF VE1 immunohistochemistry**			
Negative (n=242)	172 (71.1%)	70 (28.9%)	0.012
Positive (n=29)	14 (48.3%)	15 (51.7%)	
**MMR enzyme status**			
MMR-deficient (n=38)	21 (55.3%)	17 (44.7%)	0.050
MMR-proficient (n=232)	165 (71.1%)	67 (28.9%)	
**Modified Glasgow Prognostic Score**			
0 (n=209)	156 (74.6%)	53 (25.4%)	9.9E-5
1 (n=54)	29 (53.7%)	25 (46.3%)	
2 (n=7)	1 (14.3%)	6 (85.7%)	

Abbreviations: IQR: interquartile range, MMR: mismatch repair. P values are for Mann-Whitney or Kruskal-Wallis test.

Table S5. Correlations between serum MMP-8 and serum cytokine levels in Cohort 1

	**Unadjusted**		**Adjusted**	
	Pearson r	p value	Beta	p value
Serum IL-1ra	0.609	5.4E-13	0.545	2.6E-10
Serum IL-4	0.372	4.3E-5	0.335	1.0E-4
Serum IL-6	0.460	2.6E-7	0.333	1.9E-4
Serum IL-7	0.491	2.5E-8	0.402	5.0E-6
Serum IL-8	0.550	1.9E-10	0.433	2.5E-5
Serum IL-9	0.265	0.004	0.205	0.021
Serum IL-12(p70)	0.395	1.3E-5	0.279	0.001
Serum IFNγ	0.350	1.2E-4	0.288	0.001
Serum CXCL10	0.278	0.0026	0.212	0.021
Serum CCL2	0.340	2.1E-4	0.288	0.001
Serum CCL4	0.322	4.4E-4	0.218	0.013
Serum CCL11	0.096	0.309	0.076	0.402
Serum PDGF-BB	0.471	1.1E-7	0.279	0.001

The correlations were adjusted for tumor stage variables (T1-2 vs. T3-4; N0 vs. N1-2; M0 vs. M1), patient age, and patient gender by multiple linear regression. Abbreviations: IL: interleukin; IFN: interferon; CCL: Chemokine (C-C motif) ligand; CXCL: Chemokine (C-X-C motif) ligand; PDGF: Platelet-derived growth factor.

Table S6. Receiver operating characteristics (ROC) analysis areas under the curve (AUCs) for different continuous and categorical variables in detecting survivors from non-survivors (cancer specific survival)

Variable	Area under the curve (AUC)	95% Confidence interval	P value
**Continuous variables**			
Serum MMP-8	0.683	0.600-0.766	1.1E-5
Serum CRP	0.611	0.526-0.696	0.008
Serum albumin	0.583	0.498-0.668	0.047
Blood neutrophil count	0.599	0.518-0.681	0.017
Blood neutrophil/lymphocyte ratio	0.593	0.514-0.673	0.025
**Categorical variables**			
TNM stage (I, II, III, IV)	0.858	0.803-0.912	1.3E-17
T-class (T1, T2, T3, T4)	0.614	0.532-0.696	0.006
N-class (N0, N1, N2)	0.789	0.722-0.857	6.5E-12
M-class (M0, M1)	0.732	0.651-0.814	2.3E-8
Grade (1, 2, 3)	0.606	0.529-0.683	0.011
Lymphatic invasion (no, yes)	0.720	0.650-0.791	1.7E-7
Blood vessel invasion (no, yes)	0.662	0.577-0.747	1.2E-4
BRAF VE1 immunohistochemistry (negative, positive)	0.508	0.427-0.589	0.854
MMR status (proficient, deficient)	0.581	0.506-0.656	0.053
Immunoscore (0, 1, 2, 3, 4)	0.722	0.652-0.791	9.9E-8
Immunoscore (0-1, 2-4)	0.683	0.608-0.759	1.0E-5
Serum MMP-8 (≤100 ng/mL, >100 ng/mL)	0.647	0.567-0.728	4.0E-4
Serum CRP (≤20 mg/L, >20 mg/L)	0.626	0.540-0.711	0.003
mGPS (0, 1, 2)	0.596	0.512-0.681	0.021
mGPS (0, 1-2)	0.591	0.507-0.674	0.029
**Combination variables**			
Serum MMP-8 (≤100 ng/mL, >100 ng/mL) and Immunoscore (0-1, 2-4)	0.744	0.675-0.812	4.7E-9
Serum MMP-8 (≤100 ng/mL, >100 ng/mL) and serum CRP (≤20 mg/L, >20 mg/L)	0.686	0.605-0.767	7.9E-6
Serum MMP-8 (≤100 ng/mL, >100 ng/mL) and depth of tumor invasion (T1-2, T3-4)	0.661	0.579-0.743	1.2E-4
Serum MMP-8 (≤100 ng/mL, >100 ng/mL) and nodal metastasis (N0, N1-2)	0.782	0.716-0.847	2.3E-11
Serum MMP-8 (≤100 ng/mL, >100 ng/mL) and distant metastasis (M0, M1)	0.744	0.665-0.823	4.5E-9
Serum MMP-8 (≤100 ng/mL, >100 ng/mL) and lymphatic invasion (no, yes)	0.756	0.686-0.825	1.3E-9
Serum MMP-8 (≤100 ng/mL, >100 ng/mL) and blood vessel invasion (no, yes)	0.714	0.634-0.794	3.9E-7
Serum MMP-8 (≤100 ng/mL, >100 ng/mL) and mGPS (0, 1, 2)	0.661	0.579-0.743	1.1E-4

Table S7. Multivariate analysis of 60-month cancer-specific survival (CSS) and overall survival (OS) of CRC patients in the combined cohort

	**CSS**			**OS**		
	HR	95%CI	p value	HR	95%CI	p value
Age (<65 vs. ≥65)	1.56	0.85-2.85	0.148	1.91	1.12-3.28	0.018
Tumor invasion (T1-T2 vs. T3-T4)	0.61	0.30-1.25	0.176	0.60	0.34-1.05	0.071
Nodal metastases (N0 vs. N1-N2)	3.97	1.75-8.98	0.001	2.34	1.29-4.26	0.005
Distant metastases (M0 vs. M1)	6.21	3.18-12.12	8.8E-8	3.73	2.09-6.65	7.8E-6
Lymphatic invasion (No vs. Yes)	1.35	0.60-3.01	0.469	1.04	0.57-1.90	0.902
Grade (1-2 vs. 3)	2.08	1.28-3.38	0.003	1.96	1.33-2.89	0.001
mGPS (0 vs. 1-2)	1.08	0.57-2.05	0.823	1.45	0.86-2.46	0.162
BRAF VE1 immunohistochemistry (negative vs. positive)	2.83	1.02-7.85	0.046	1.36	0.56-3.33	0.495
MMR status (proficient vs. deficient)	0.17	0.02-1.33	0.091	0.80	0.32-1.98	0.627
Immunoscore (0-1 vs. 2-4)	0.48	0.26-0.90	0.022	0.46	0.28-0.77	0.003
Serum MMP-8 (≤100 ng/mL vs. >100 ng/mL)	2.04	1.15-3.62	0.015	1.60	0.98-2.61	0.062

CSS: n=264; median follow-up time 60.0 months (IQR 36.5-60.0); 57 (21.6%) events; 7 (2.6%) cases excluded from the analysis because of missing values.
OS: n=266; median follow-up time 60.0 months (IQR 36.5-60.0); 81 (30.5%) events; 5 (1.8%) cases excluded from the analysis because of missing values.
Abbreviations: CI: confidence interval, HR: hazard ratio.
